# Functional and Neurotrophic Outcomes After Physical Therapy and Injection-Based Treatments for Rotator Cuff Tendinopathy: The FORTIS Pilot Randomized Trial

**DOI:** 10.1177/23259671261468532

**Published:** 2026-08-26

**Authors:** Natalia Pawłuś, Szymon Turkiewicz, Jędrzej Lesman, Agata Gabryelska, Marcin Domżalski, Michał Kanak

**Affiliations:** † Department of Orthopaedics and Traumatology, Medical University of Lodz, University Clinical Hospital No. 2 of the Medical University of Lodz, Lodz, Poland; ‡ Department of Sleep Medicine and Metabolic Disorders, Medical University of Lodz, Lodz, Poland; Investigation performed at the Department of Orthopaedics and Traumatology, Medical University of Lodz, University Clinical Hospital No. 2 of the Medical University of Lodz, Lodz, Poland

**Keywords:** rotator cuff tendinopathy, physical therapy, platelet-rich plasma, suprascapular nerve block, sleep disturbance, brain-derived neurotrophic factor

## Abstract

**Background:**

Rotator cuff tendinopathy (RCT) causes chronic shoulder pain, functional limitation, and sleep disturbance. Although physical therapy (PT) and injection-based interventions are widely used, their comparative clinical effects and neurotrophic mechanisms remain insufficiently defined.

**Purpose:**

To compare clinical and neurotrophic outcomes of PT alone versus PT combined with injection-based interventions in patients with chronic RCT.

**Study Design:**

Randomized controlled trial; Level of evidence, 2.

**Methods:**

A total of 29 patients (29 shoulders) with MRI-confirmed supraspinatus tendinopathy and sleep disturbance (Pittsburgh Sleep Quality Index score ≥5) were randomized to 1 of 4 groups: PT (n = 7), PT with subacromial steroid injection (PT + SSI; n = 6), PT with suprascapular nerve block (PT + SSNB; n = 8), or PT with platelet-rich plasma injection (PT + PRP; n = 8). All participants completed a standardized 12-week PT program. Clinical outcomes were assessed at baseline (time zero [T0]), 1 month (T1), and 3 months (T3). Serum brain-derived neurotrophic factor (BDNF) was measured at T0 and T1. Wilcoxon and Kruskal-Wallis tests assessed within- and between-group differences.

**Results:**

All groups demonstrated clinical improvement, most consistently in pain, insomnia severity, shoulder function, and cognitive performance. PT + PRP improved across pain, insomnia, and shoulder function outcomes (*P*≤ .03). PT showed significant improvements in pain, insomnia severity, shoulder function, and processing speed (all *P*≤ .04). PT + SSI demonstrated early pain reduction at 1 month (*P* = .03), whereas PT + SSNB showed selective improvements in sleep quality (*P* = .02) and executive function (*P* = .04). No significant between-group differences were observed in serum BDNF levels. Only PT demonstrated significant correlations between BDNF changes and reductions in pain and insomnia severity (*P* < .01).

**Conclusion:**

Our study demonstrated that PT alone was associated with coordinated short-term clinical improvement and neurotrophic correlates, supporting its role as a core component of nonoperative management of RCT. Injection-based adjuncts produced selective, time-limited effects. These findings suggest that structured PT should remain the cornerstone of nonoperative management, while injections may serve as adjuncts to facilitate short-term symptom relief and rehabilitation engagement rather than as stand-alone treatments.

**Registration:**

NCT06631976 (ClinicalTrials.gov identifier).

Shoulder pain is one of the most common musculoskeletal complaints and a major cause of functional limitation and sleep disturbance, with rotator cuff (RC) pathology accounting for approximately 65% to 70% of shoulder-related symptoms.^40,45^ Up to 93% of patients with RC disorders report clinically relevant sleep impairment, frequently cited as the primary reason for seeking medical care.^6,12,23,26^ A recent scoping review highlighted that nocturnal shoulder pain may reflect a wide range of underlying conditions, from referred or systemic disease to musculoskeletal pathology, with RC conditions being the most common underlying cause.^40^

The interaction between chronic pain and sleep disturbance involves neurobiological mechanisms beyond behavioral factors, with brain-derived neurotrophic factor (BDNF) emerging as a potential mediator linking pain modulation and sleep regulation.^11^ Circulating BDNF levels reflect central neurotrophic activity and have been implicated in synaptic plasticity and central sensitization.^15,30,51^ In humans, both acute sleep deprivation and chronic insomnia are associated with altered BDNF concentrations,^10,17^ and the modulation of BDNF signaling has been shown to influence pain sensitivity.^52^ Altered serum BDNF levels have also been reported in chronic musculoskeletal pain syndromes.^19,53^ However, the role of BDNF in localized shoulder pathologies, including RC tendinopathy (RCT), remains unclear.

Therefore, the purpose of this study is to compare clinical and neurotrophic outcomes of physical therapy (PT) alone versus PT combined with injection-based interventions in patients with chronic RCT. We hypothesized that improvements in sleep, function, and pain would be associated with changes in circulating BDNF, reflecting neuroplastic adaptations that accompany clinical recovery.

## Methods

### Trial Design

This pilot, randomized, parallel-group clinical trial was conducted at the Department of Orthopaedics and Traumatology, University Clinical Hospital No. 2, Medical University of Lodz (June 2024-October 2025). Participants were randomized using sealed, opaque, sequentially numbered envelopes prepared by an independent administrator (M.D.) and assigned to 1 of 4 nonoperative treatment groups in a single-blind manner. An independent administrator (J.L.) generated the random allocation sequence using a computer-generated randomization list (simple randomization method). The personnel responsible for participant enrollment and intervention assignment had no access to the random allocation sequence.

The enrolling physician (M.D.), laboratory investigators (S.T. and A.G.), and statistician (J.L.) were blinded to group allocation, whereas treating clinicians (N.P. and M.K.) were not. Participants were not blinded to group allocation. Blinding was achieved by concealing group allocation from personnel involved in eligibility assessment, laboratory analyses, and statistical evaluation.

As this was a pilot randomized trial, no formal a priori sample size calculation was performed. The final sample size was determined based on feasibility considerations related to the laboratory capacity. A total of 29 participants completed the study and were included in the final analysis. No interim analyses were planned or conducted, and no stopping guidelines were predefined.

Ethical approval was obtained, and the study was registered at clinicaltrials.gov on June 4, 2024. Reporting followed the CONSORT (Consolidated Standards of Reporting Trials) guidelines,^21^ as part of the FORTIS (Functional Outcomes after Rotator cuff Therapy and Injection Strategies) project.

Patients and members of the public were not involved in the design, conduct, analysis, or reporting of this pilot randomized trial. The trial was completed as planned and was not stopped early.

### Participants and Eligibility Criteria

Patients presenting with nontraumatic shoulder pain and sleep disturbance related to partial-thickness RCT were screened for inclusion at a tertiary orthopaedic outpatient clinic. Eligible participants were allocated to 1 of 4 treatment groups: PT, PT with subacromial steroid injection (PT + SSI), PT with suprascapular nerve block (PT + SSNB), or PT with platelet-rich plasma injection (PT + PRP).

Inclusion criteria were written informed consent; magnetic resonance imaging (MRI)–confirmed symptomatic partial-thickness supraspinatus tear <1 cm in length and width and <50% of tendon thickness; no prior biological treatment or surgical intervention to the affected shoulder; sleep disturbance lasting ≥4 weeks with a Pittsburgh Sleep Quality Index (PSQI) score ≥5 attributed to shoulder pain; day- and night-time shoulder pain; age of 40 to 70 years; and body mass index (BMI) <30 kg/m^2^.

Exclusion criteria included other concomitant painful conditions; bilateral symptomatic shoulder pain; prior injection or surgical treatment of the affected shoulder; active or previous malignancy; chronic inflammatory, neurological, or psychiatric disorders; history of cardiovascular or cerebrovascular events; conditions impairing tendon healing (type 2 diabetes, osteoporosis, hypercholesterolemia); sleep disorders unrelated to shoulder pain; shift work; intercontinental travel within 2 weeks before enrollment; alcohol or drug abuse; nicotine dependence or passive smoking; and use of hypnotic, sleep-modulating, antidepressant, antipsychotic, or anxiolytic medications.

### Protocol and Assessment Timeline

The study included a baseline assessment (time zero [T0]) with subsequent follow-up visits at 1 month (T1) and 3 months (T3).

At baseline, eligibility was confirmed by an orthopaedic physician (M.D.) based on medical history, physical examination to verify supraspinatus tendinopathy and exclude other shoulder pathology, and MRI review. Participants then completed validated instruments assessing pain intensity (Numeric Rating Scale [NRS]^24^; Laitinen Pain Scale [LPS]^28^), sleep quality (PSQI^5^), insomnia severity (Athens Insomnia Scale [AIS]^14,47^), cognitive performance (Montreal Cognitive Assessment [MoCA]^16,32,37^), processing speed (Trail Making Test Part A [TMT-A]^3,20^), executive function (Trail Making Test Part B [TMT-B]^3,20^), and shoulder function (American Shoulder and Elbow Surgeons [ASES] score^34^; Simple Shoulder Test [SST]^18^; and Constant-Murley score [CMS]^41^).

At each study visit (T0, T1, and T3), venous blood samples were collected into serum tubes for BDNF analysis. Blood sampling and questionnaire-based assessments were performed in the morning, prior to any intervention or clinical evaluation.

The study had 3 primary outcomes: pain intensity (NRS), sleep disturbance (PSQI), and shoulder function (ASES), analyzed as changes from T0 to T3. Secondary outcomes included LPS, AIS, SST, CMS, MoCA, TMT-A, TMT-B, and serum BDNF.

### Interventions

All injection-based interventions were performed by a board-certified orthopaedic surgeon (M.K.) with experience in ultrasound-guided musculoskeletal procedures, using real-time ultrasound guidance and standard aseptic technique. A standardized home-based PT program was initiated within 24 hours after each intervention.

Adverse events were monitored throughout the study and recorded at each follow-up visit. They were assessed systematically at each study visit using active participant inquiry and clinical evaluation. Minor and transient adverse events, including increased local warmth of the shoulder and tenderness at the injection site, were observed and resolved spontaneously within 24 to 48 hours.

Concomitant care was standardized across all groups. Participants were instructed to avoid analgesic and anti-inflammatory medications during the study period because of their potential influence on pain and sleep assessments, to refrain from strenuous physical activity until the initiation of the PT program, and to follow a uniform home-based PT protocol.

#### PRP Injection

In the PT + PRP group, autologous venous blood was processed using the Tropocells system (Estar Technologies Ltd) to obtain leukocyte-rich, nonactivated PRP. A final volume of 2 to 3 mL PRP was injected into the supraspinatus tendon under ultrasound guidance using an in-plane approach with the patient in the Crass position.^49^ PRP preparation was reported in accordance with the Minimum Information for Studies Evaluating Biologics in Orthopaedics (MIBO) recommendations.^36^

#### Suprascapular Nerve Block

In the PT + SSNB group, 5 mL of 2% lidocaine was administered under ultrasound guidance around the suprascapular nerve along its course between the suprascapular notch and the spinoglenoid notch, in accordance with the recommendations of the American Society of Regional Anesthesia and Pain Medicine.^2^

#### Subacromial Steroid Injection

In the PT + SSI group, 1 mL of corticosteroid suspension (40 mg/mL methylprednisolone acetate equivalent) was injected into the subacromial-subdeltoid bursa under ultrasound guidance. The Crass position was used to enhance visualization of the subacromial space.^49^

#### PT Protocol

The PT program was prescribed and reviewed by a single licensed physical therapist (N.P.) with clinical experience in shoulder rehabilitation.

Participants followed a standardized home-based PT program supported by best-practice advice recommendations.^22^ No supervised PT sessions were provided between visits. At T0, participants received individualized instruction on correct exercise execution. At T1 and T3, the program was reviewed and progressed during additional individual sessions, including the introduction of new exercises and advancement of loading as tolerated.

Compliance with the PT protocol was monitored throughout the intervention period. During follow-up assessments, the PT program was reviewed and progressed, and the prescribed home-based PT program was documented and provided to participants. During these individual sessions, the physical therapist verified correct exercise technique, ensured that each participant was able to perform the prescribed exercises properly, and addressed any uncertainties regarding exercise execution. Participants received instructions in printed form and as short video recordings. Adherence to the home-exercise program was additionally supported and verified through regular telephone follow-up, typically performed once weekly.

The program comprised a uniform exercise framework across all groups, including active range-of-motion exercises, isometric RC loading, and progressive resistance training using elastic bands and lightweight dumbbells. Core components included isometric external and internal rotation in adduction and abduction, scapular retraction exercises, and graded shoulder elevation tasks (eg, scaption, forward flexion, and abduction), progressing from side-lying to upright positions.

Limited individual tailoring was permitted based on pain tolerance, range of motion, and functional capacity, involving adjustments in resistance, movement range, and repetitions while preserving the overall program structure. Progression was symptom guided: range of motion was increased when pain-free, resistance was advanced gradually, and repetitions progressed up to approximately 12 per set as tolerated.

Participants were instructed to maintain exercise-related pain at ≤3 out of 10 on the NRS, perform a 5- to 10-minute warm-up before each session, exercise ≥4 times per week, and contact the investigator in case of uncertainty regarding the technique or adverse symptoms. No fixed session duration was prescribed.

### Biochemical Analysis

Venous blood samples were collected at T0, T1, and T3 into serum tubes with clot activator, centrifuged at 3500 *g* for 15 minutes at 4°C, and the obtained serum was aliquoted and stored at −80°C until analysis. Serum BDNF concentrations were measured using a commercially available enzyme-linked immunosorbent assay kit (Human BDNF ELISA; FineTest). Absorbance was read at 450 nm using a microplate reader (BioTek 800 TS; Agilent Technologies). All analyses were performed in accordance with the manufacturer's guidelines.

### Statistical Analysis

Continuous variables were summarized as medians with interquartile ranges. Normality of distributions was assessed using the Shapiro-Wilk test. Given the exploratory and pilot nature of the study, nonparametric statistical methods were applied. Within-group comparisons over time were performed using the Wilcoxon signed-rank test, and between-group differences were assessed using the Kruskal-Wallis test. Adverse events were summarized descriptively.

Statistical analyses were conducted using available data from participants who completed the respective assessments. No imputation of missing data was performed. All tests were 2-tailed, and statistical significance was set at *P* < .05. Statistical analyses were performed using Python (Version 3.11.4) and Statistica (Version 13.3; TIBCO Software Inc). No prespecified subgroup or sensitivity analyses were conducted; exploratory post hoc analyses assessed associations between serum BDNF changes and clinical outcomes.

## Results

Results are presented for the co-primary outcomes of sleep disturbance, pain, and shoulder function, as well as the secondary outcomes of cognitive performance and serum BDNF levels.

**Figure 1.**
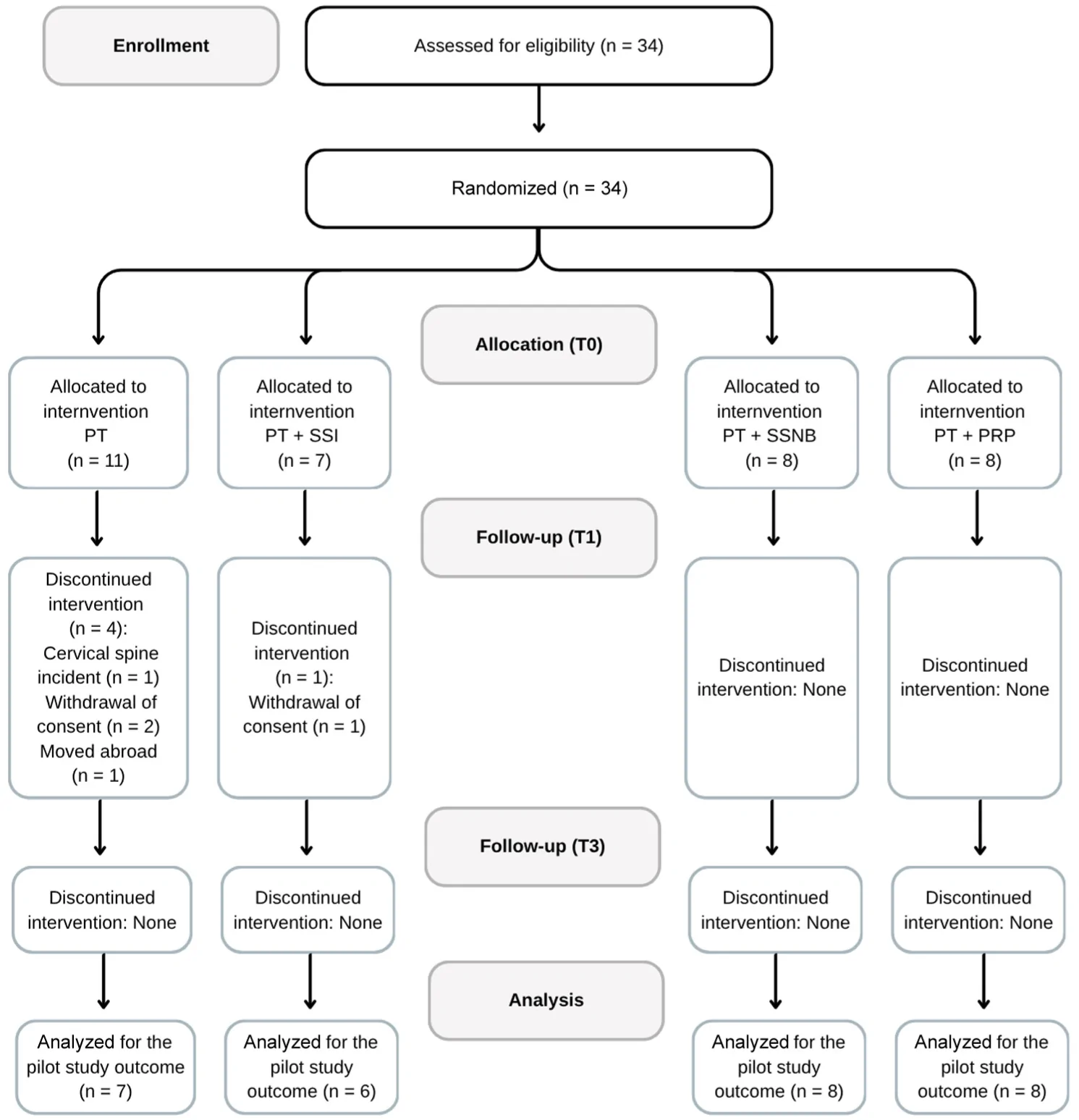
CONSORT (Consolidated Standards of Reporting Trials) 2010 flow diagram. PRP, platelet-rich plasma; PT, physical therapy; SSI, subacromial steroid injection; SSNB, suprascapular nerve block; T0-T3, time zero (baseline)-time 3 (months).

A total of 34 participants (34 shoulders) were randomized into 4 intervention groups. Of these, 29 participants (29 shoulders) completed the study and were included in the final analysis. Details of participant flow, including group-specific losses and reasons for discontinuation, are presented in Figure 1, prepared in accordance with CONSORT 2010 flow diagram recommendations.^21^

Participants were allocated into 4 groups: PT (n = 7), PT + SSI (n = 6), PT + SSNB (n = 8), and PT + PRP (n = 8). Minor differences in group size reflect the pilot nature of the study. No serious adverse events were observed in any group.

### Baseline Demographic, Anthropometric, and Clinical Characteristics

Baseline demographic and anthropometric characteristics, as well as baseline clinical, sleep, cognitive, and functional outcome scores, are presented in AppendixTables A1 and A2. The study groups were comparable with respect to age, sex distribution, BMI, and all baseline outcome measures, with no significant between-group differences observed at baseline (all *P* > .05).

### Changes in Clinical Outcomes

**Figure 2.**
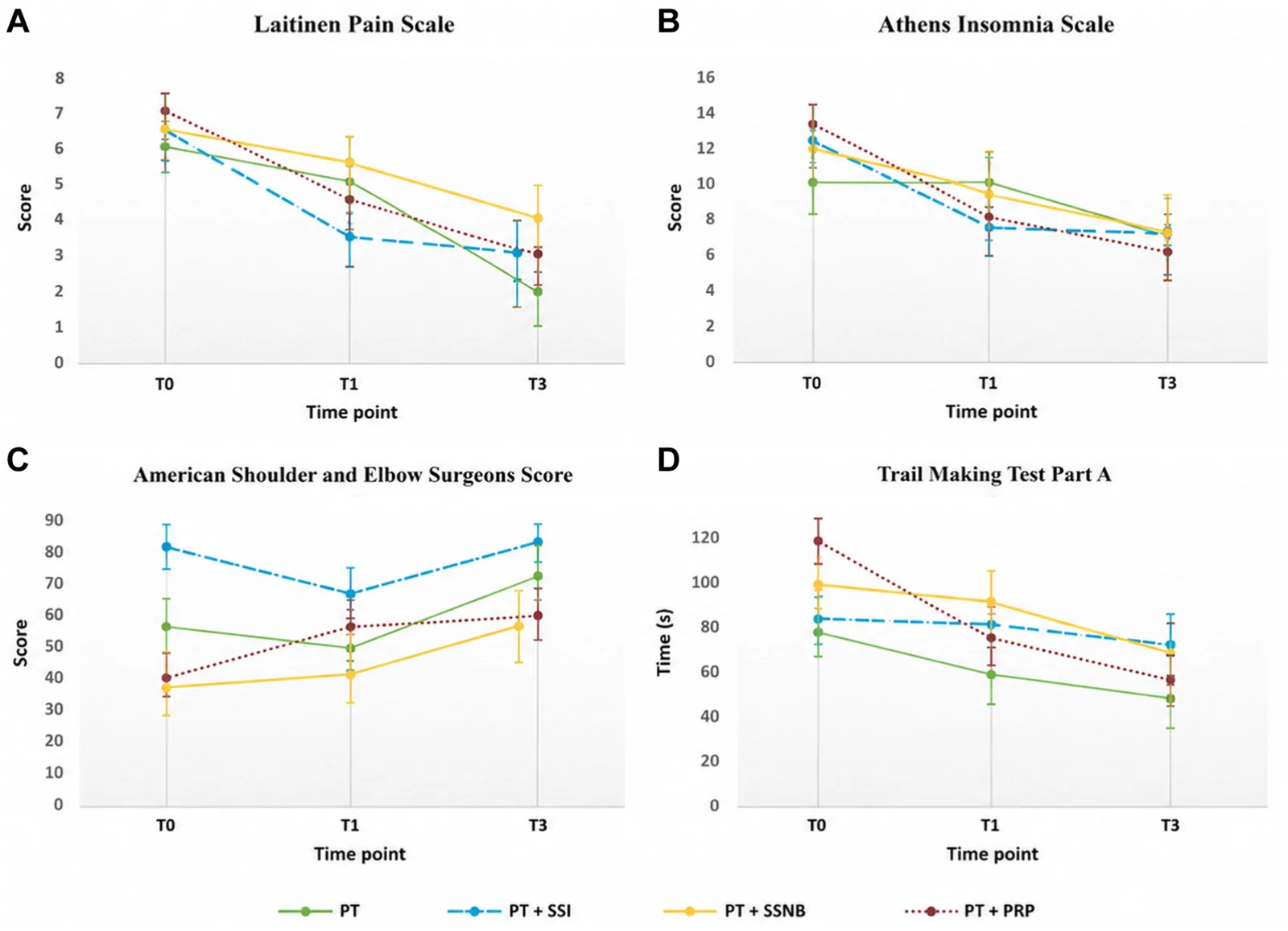
Median changes across study groups at T0, T1, and T3. Lines represent median values; error bars represent IQRs. (A) Laitinen Pain Scale showing the median decrease in shoulder pain intensity; (B) Athens Insomnia Scale showing the median decrease in insomnia severity; (C) American Shoulder and Elbow Surgeons score showing the median increase in functional performance; (D) Trail Making Test Part A showing the median decrease in completion time, corresponding to faster processing speed. PRP, platelet-rich plasma; PT, physical therapy; SSI, subacromial steroid injection; SSNB, suprascapular nerve block; T0-T3, time zero (baseline)-time 3 (months).

Across all treatment groups, clinical outcomes improved over time between T0, T1, and T3. The most consistent and statistically significant improvements were observed in pain (LPS), insomnia severity (AIS), shoulder function (ASES), and processing speed (TMT-A) (Figure 2), with additional outcomes showing similar but less pronounced trends (AppendixTable A3). The magnitude and temporal pattern of improvement varied across groups.

The PT + PRP group showed the most consistent and multidimensional response, with significant gains in pain (LPS: T0-T3, *P* = .008), insomnia severity (AIS: T0-T3, *P* = .03), and shoulder function (ASES: *P* = .01; CMS: *P* = .01; SST: *P* = .008). These effects developed progressively and were most evident at 3 months.

The PT group also showed a favorable trajectory, with significant reductions in pain (LPS: *P* = .03) and insomnia severity (AIS: *P* = .03), accompanied by improvements in shoulder function (ASES: *P* = .04) and processing speed (TMT-A: *P* = .04), primarily emerging between 1 and 3 months.

The PT + SSI group exhibited an early but less sustained response, with significant pain relief at 1 month (NRS: *P* = .03) and concurrent improvements in shoulder function (ASES: *P* = .03) and processing speed (TMT-A: *P* = .03), without further progression at 3 months.

The PT + SSNB group showed selective improvements, mainly in sleep quality (PSQI: *P* = .02) and executive function (TMT-B: *P* = .04), while changes in pain intensity and shoulder-specific outcomes were modest or not significant.

### Serum BDNF Levels Over Time

The serum BDNF concentrations are summarized in AppendixTable A4. Across all study groups, BDNF levels showed a mild upward trend between T0 and T3, most evident in the PT + PRP and PT + SSNB groups. Within-group comparisons between T0 and T1 were not statistically significant (all *P* > .05), and no between-group differences were observed at T1 (Kruskal-Wallis test; *P* = .38). Analyses at T3 were conducted using available data.

### Association Between BDNF Changes and Clinical Outcomes

**Figure 3.**
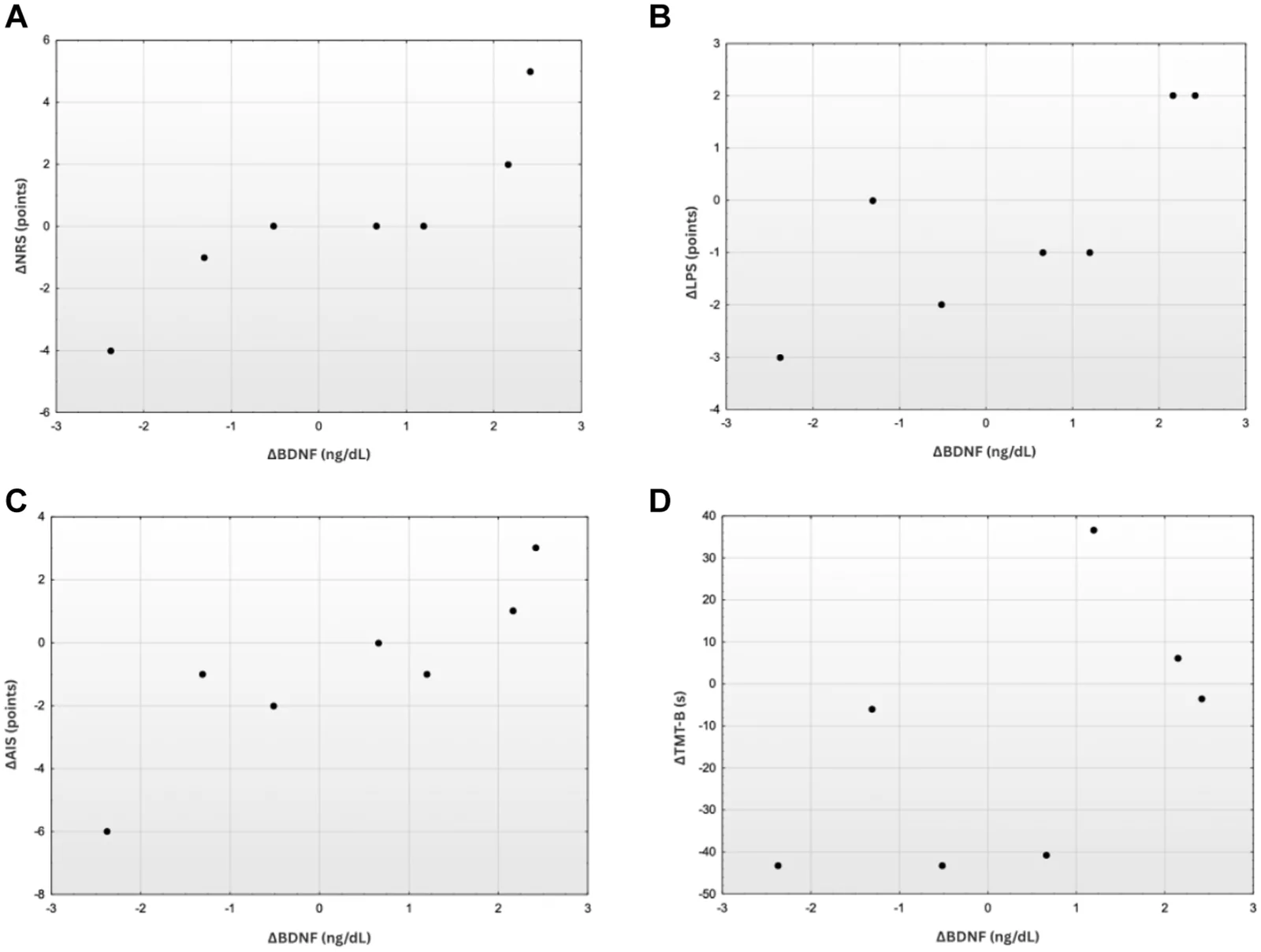
Correlations between changes in serum BDNF concentration (ΔBDNF; ng/dL) and clinical outcomes (ΔT1-T0) in the PT group. (A) ΔNRS; (B) ΔLPS; (C) ΔAIS; (D) ΔTMT-B. Each point represents 1 participant; Δ indicates the change between T1 and T0. AIS, Athens Insomnia Scale; BDNF, brain-derived neurotrophic factor; LPS, Laitinen Pain Scale; NRS, Numeric Rating Scale; TMT-B, Trail Making Test, Part B.

Changes in serum BDNF concentration (ΔT1-T0) were analyzed in relation to changes in pain, sleep, cognitive performance, and functional scores (AppendixTable A5; Figure 3).

Significant associations were observed exclusively in the PT group. In this group, increases in serum BDNF were strongly and negatively correlated with reductions in pain and insomnia severity, particularly ΔNRS (*P* < .01), ΔLPS (*P* = .04), and ΔAIS (*P* < .01), indicating that greater BDNF increases were associated with larger clinical improvements (Figure 3, A-C). A near-significant association was also observed for executive function assessed by ΔTMT-B (*P* = .05) (Figure 3D).

No significant correlations were found between ΔBDNF and shoulder-specific functional outcomes (ASES, SST, CMS) in the PT group. Similarly, no significant associations between ΔBDNF and any clinical outcomes were detected in the PT + SSI, PT + SSNB, or PT + PRP groups (*P* > .05).

## Discussion

### Overview of Principal Findings

Our study demonstrated that all nonoperative strategies were associated with short-term clinical improvement, although the magnitude and timing of response differed across interventions. Pain-related improvements were most evident after PT + SSI and PT + PRP, including reductions in NRS pain after PT + SSI from T0 to T1 (*P* = .03) and after PT + PRP from T0 to T3 (*P* = .02), while LPS scores improved significantly after PT + PRP from T0 to T3 (*P* = .008). Sleep outcomes improved selectively, with AIS improvements from T0 to T3 in the PT + SSI, PT + SSNB, and PT + PRP groups (*P* = .03, *P* = .02, and *P* = .03, respectively). Shoulder function also improved, and between-group differences at T3 were observed for ASES, CMS, and SST scores (*P* = .04, *P* = .047, and *P* = .04, respectively). Notably, BDNF-related associations were observed only in the PT group, suggesting a potential exercise-linked neurotrophic pattern that is discussed further below.

In line with current evidence indicating that nonoperative treatment remains the cornerstone of management for RC-related shoulder pain, the present findings support structured PT as a primary contributor to early clinical improvement.^22,27^ PT administered alone or combined with SSI, SSNB, or PRP was associated with short-term improvements in pain and shoulder function across all groups, consistent with prior reports on the effectiveness of individualized rehabilitation programs.^22,27^

Although previous studies suggest that injection-based treatments may provide early symptom relief, with PRP potentially exerting more sustained effects beyond the early recovery phase,^4,40,41,48^ adjunctive injections in the present pilot trial were associated with heterogeneous responses and did not clearly modify the early clinical trajectory within the 3-month follow-up. From an orthopaedic perspective, these findings reinforce the importance of progressive loading and neuromuscular retraining during early recovery in RCT, with injections serving as adjuncts to facilitate rehabilitation in selected patients.

To our knowledge, this is the first clinical study to assess serum BDNF dynamics in the context of both rehabilitation-only and injection-assisted treatment strategies for RCT.

### Exercise-Induced BDNF Signaling and Pain Modulation

Physical activity is a recognized modulator of BDNF, with exercise-related increases in circulating BDNF associated with activity-dependent neuroplastic adaptations and modulation of pain-processing pathways.^8,9,38,48^ The magnitude of this response depends on exercise intensity, duration, and cumulative load, with sustained activity eliciting more pronounced neurotrophic effects.^8,9,48^ Exercise-induced BDNF signaling has been linked to synaptic plasticity and descending pain modulation, particularly in conditions involving altered central pain processing.^29,33^

Task-specific PT may therefore act not only as a mechanical intervention targeting tendon loading and neuromuscular control, but also as a biological stimulus engaging movement-dependent neuroplastic mechanisms.^31,39^ Sustained muscle activation and proprioceptive input provide continuous afferent signaling capable of influencing nociceptive processing, especially during early rehabilitation phases characterized by pain modulation rather than structural tissue remodeling.^31,39^

In the present study, significant associations between increases in serum BDNF concentration and improvements in pain intensity were observed exclusively in the PT group, with no comparable relationship in injection-based groups. This pattern suggests that exercise-driven neurotrophic engagement is most closely aligned with clinical improvement when PT is the isolated therapeutic stimulus. By contrast, injection-based treatments may reduce pain primarily through local anti-inflammatory and peripheral neural mechanisms, without engaging movement-dependent neurotrophic pathways associated with increased serum BDNF levels.^33,46^

Overall, these findings suggest that circulating BDNF may reflect exercise-related neuroplastic engagement more clearly than clinical response across heterogeneous treatment strategies.

### The Pain-Sleep-Neurotrophic Axis in RCT

BDNF plays an important role in sleep and circadian regulation, and reduced BDNF expression is associated with shorter rapid eye movement sleep duration, lower sleep efficiency, and alterations in sleep architecture.^1,10,17^ Disturbed sleep, in turn, has been linked to impaired recovery and increased symptom burden in chronic musculoskeletal conditions, including RC-related shoulder pain.^10,17^

Within this framework, an interconnected pain-sleep-neurotrophic axis has been proposed, in which sleep disturbance, reduced neurotrophic signaling, and heightened pain sensitivity interact in a bidirectional and potentially self-reinforcing cycle.^4,17,44^ Altered neurotrophic activity has been linked to impaired sleep regulation and altered pain processing, which may further disrupt sleep and perpetuate pain-related disability.^29,33,44^

In the present study, improvements in insomnia severity were most pronounced in the PT group and were accompanied by significant associations between changes in serum BDNF levels and sleep outcomes (AIS). Although sleep-related outcomes improved across all treatment groups, neurotrophic-clinical coupling was observed exclusively when PT constituted the isolated therapeutic stimulus. This exploratory finding suggests that exercise-based PT may be more closely associated with sleep-related neuroplastic mechanisms than injection-based treatments.^13,35^

Importantly, the absence of BDNF–clinical coupling in the injection-assisted groups should not be interpreted as a suppressive effect of the injections on neuroplastic processes. Injection-based treatments may effectively reduce pain and facilitate participation in rehabilitation.^29,33^ In the SSNB group, pain relief was accompanied by improvements in sleep quality and executive function, suggesting a clinically relevant but potentially different mechanism of benefit. However, the primary effects of injection-based interventions are likely mediated through peripheral anti-inflammatory or neural pathways rather than sustained, movement-dependent neurotrophic activation.^29,33,46^ In this context, injections may primarily modulate peripheral nociceptive input or local inflammatory signaling, without providing the repeated afferent stimulation required to promote central neuroplastic adaptation.^29,46^

Moreover, many studies reporting favorable outcomes after injection-based interventions focus primarily on pain and function, without concurrent assessment of sleep quality or neurotrophic markers, limiting mechanistic interpretation.^7,22,25,42,43,50^ Clinically, these findings underscore the importance of addressing sleep quality and activity-dependent rehabilitation as integral components of nonoperative management of RCT.

### Limitations

This pilot study was limited by a small sample size, partial blinding, and incomplete BDNF data at 3 months, which reduced the precision of effect estimates and limited assessment of longer-term neurotrophic changes. Full participant blinding was not feasible because of the ethical and practical constraints related to injection-based interventions. As no a priori power analysis was performed, the variability observed in this cohort will be used to inform sample size estimation for a future adequately powered randomized trial.

Adequately powered randomized trials with longer follow-up periods and predefined adherence monitoring are needed to determine whether injection-based treatments provide clinically meaningful benefits beyond structured PT alone. Further studies should also aim to identify clinical and structural patient characteristics associated with better responses to specific adjunctive interventions.

## Conclusion

Our study demonstrated that PT alone was associated with coordinated short-term clinical improvement and neurotrophic correlates, supporting its role as a core component of nonoperative management of RCT. Injection-based adjuncts produced selective, time-limited effects and may facilitate short-term symptom relief and rehabilitation engagement rather than serve as stand-alone treatments. Larger studies with longer follow-up are warranted to confirm these preliminary findings and further clarify the role of BDNF in musculoskeletal rehabilitation.

## Appendix

**Table A1:** Baseline Characteristics of Study Participants*^*a*^*

Variable	PT	PT + SSI	PT + SSNB	PT + PRP
Age, y	53.0 (50.0-56.0)	51.5 (50.25-55.0)	62.4 (55.25-69.5)	53.0 (49.75-57.75)
Sex, M/F, %	71.4/28.6	66.7/33.3	62.5/37.5	25.0/75.0
BMI, kg/m^2^	26.8 (24.4-29.15)	27.3 (25.59-28.94)	27.4 (25.96-28.76)	25.9 (24.37-27.50)
Higher education	4 (57.1)	2 (33.3)	0 (0.0)	2 (25.0)
Occupational status (employed/retired)	100.0/0.0	83.3/16.7	50.0/50.0	37.5/62.5
Urban residence, ≥20,000 inhabitants	57.1	50.0	50.5	37.5

a Values are presented as median (IQR), n, or %. BMI, body mass index; F, female; M, male; PRP, platelet-rich plasma; PT, physical therapy; SSI, subacromial steroid injection; SSNB, suprascapular nerve block.

**Table A2:** Clinical Outcome Scores Across Study Groups at Time Zero*^*a*^*

Variable	PT	PT + SSI	PT + SSNB	PT + PRP
NRS	5.0 (4.0-6.0)	8.0 (5.8-8.0)	7.5 (6.5-9.0)	6.0 (5.0-7.3)
LPS	6.0 (5.0-7.0)	6.5 (6.0-7.0)	6.5 (6.0-8.3)	7.0 (5.8-8.0)
PSQI	8.0 (6.0-9.0)	10.5 (7.0-12.5)	6.5 (6.0-9.0)	8.5 (7.3-10.5)
AIS	10.0 (8.5-12.5)	12.5 (10.5-13.8)	12.0 (9.0-14.5)	13.5 (10.0-16.0)
MoCA	28.0 (27.0-29.5)	22.0 (18.0-24.5)	25.5 (23.0-28.5)	28.5 (25.8-30.0)
TMT-A (s)	32.2 (28.0-54.9)	33.5 (28.7-40.1)	39.2 (29.8-50.0)	30.7 (29.7-48.9)
TMT-B (s)	76.6 (61.9-87.2)	82.9 (72.0-104.0)	98.5 (91.5-113.6)	117.0 (91.5-185.0)
ASES	56.7 (44.9-61.5)	43.3 (39.1-55.0)	37.5 (25.0-45.0)	40.0 (32.0-54.0)
SST	6.0 (3.5-8.0)	5.0 (1.5-7.8)	3.5 (2.0-5.3)	4.5 (3.8-6.3)
CMS	42.0 (37.5-46.0)	50.5 (41.5-52.0)	28.0 (26.0-31.0)	35.5 (31.0-36.5)

a Values are presented as median (IQR). AIS, Athens Insomnia Scale; ASES, American Shoulder and Elbow Surgeons; CMS, Constant-Murley score; LPS, Laitinen Pain Scale; MoCA, Montreal Cognitive Assessment; NRS, Numeric Rating Scale; PRP, platelet-rich plasma; PSQI, Pittsburgh Sleep Quality Index; PT, physical therapy; SSI, subacromial steroid injection; SSNB, suprascapular nerve block; SST, Simple Shoulder Test; TMT-A, Trail Making Test, Part A; TMT-B, Trail Making Test, Part B.

**Table A3:** Changes in Clinical Outcomes Between T0 and T3*^*a*^*

Scale	Group	*P*			Scale	Group	*P*		
		T0-T1	T1-T3	T0-T3			T0-T1	T1-T3	T0-T3
NRS	PT	n.s.	n.s.	n.s.	LPS	PT	n.s.	**.02**	**.03**
	PT + SSI	**.03**	n.s.	n.s.		PT + SSI	n.s.	n.s.	n.s.
	PT + SSNB	n.s.	n.s.	n.s.		PT + SSNB	n.s.	n.s.	n.s.
	PT + PRP	n.s.	n.s.	.02		PT + PRP	**.03**	**.03**	**.008**
PSQI	PT	n.s.	n.s.	n.s.	AIS	PT	n.s.	n.s.	n.s.
	PT + SSI	n.s.	n.s.	n.s.		PT + SSI	**.03**	n.s.	**.03**
	PT + SSNB	n.s.	n.s.	**.02**		PT + SSNB	**.03**	n.s.	**.02**
	PT + PRP	n.s.	n.s.	n.s.		PT + PRP	*.05*	n.s.	**.03**
TMT-A	PT	**.04**	n.s.	**.04**	TMT-B	PT	.24	.89	.50
	PT + SSI	**.03**	n.s.	*.05*		PT + SSI	.75	.07	.34
	PT + SSNB	n.s.	n.s.	n.s.		PT + SSNB	.40	**.04**	**.04**
	PT + PRP	n.s.	n.s.	n.s.		PT + PRP	.07	.26	.07
MoCA	PT	n.s.	n.s.	n.s.	ASES	PT	n.s.	**.04**	**.04**
	PT + SSI	n.s.	n.s.	n.s.		PT + SSI	**.03**	n.s.	n.s.
	PT + SSNB	n.s.	n.s.	**.03**		PT + SSNB	n.s.	n.s.	*.05*
	PT + PRP	n.s.	n.s.	n.s.		PT + PRP	**.04**	n.s.	**.01**
SST	PT	n.s.	n.s.	n.s.	CMS	PT	n.s.	n.s.	n.s.
	PT + SSI	**.03**	n.s.	n.s.		PT + SSI	n.s.	n.s.	n.s.
	PT + SSNB	n.s.	n.s.	n.s.		PT + SSNB	n.s.	n.s.	n.s.
	PT + PRP	**.01**	n.s.	**.008**		PT + PRP	**.02**	.06	**.01**

a Data are presented as median (IQR). *P* values correspond to within-group comparisons across time points (T0-T1, T1-T3, T0-T3) using the Wilcoxon signed-rank test. Bold values indicate significant results (*P* < .05); italicized values indicate marginally significant results (*P* = .05). AIS, Athens Insomnia Scale; ASES, American Shoulder and Elbow Surgeons; CMS, Constant-Murley score; LPS, Laitinen Pain Scale; MoCA, Montreal Cognitive Assessment; NRS, Numeric Rating Scale; n.s., not significant; PRP, platelet-rich plasma; PSQI, Pittsburgh Sleep Quality Index; PT, physical therapy; SSI, subacromial steroid injection; SSNB, suprascapular nerve block; SST, Simple Shoulder Test; T0-T3, time zero (baseline)-time 3 (months); TMT-A, Trail Making Test, Part A; TMT-B, Trail Making Test, Part B.

**Table A4:** Serum BDNF Levels at T1, and T3 Across Study Groups, With Within-Group Changes Between T0 and T1*^*a*^*

Group	T0 Median	T1 Median	T3 Median	T0-T1, *P*
PT	0.86 (0.5-1.6)	2.68 (1.0-3.1)	2.50 (1.2-3.9)	.37
PT + SSI	0.71 (0.1-3.6)	1.08 (0.6-3.0)	1.38 (1.0-2.2)	.68
PT + SSNB	0.42 (0.2-1.8)	0.61 (0.2-0.9)	1.62 (0.8-3.2)	.45
PT + PRP	1.24 (0.4-1.8)	0.99 (0.6-1.9)	1.31 (0.7-3.2)	.72

a Data are expressed as median (25th-75th percentile). Serum BDNF levels are shown in ng/dL. *P* values correspond to within-group comparisons between T0 and T1 using the Wilcoxon signed-rank test. BDNF, brain-derived neurotrophic factor; PRP, platelet-rich plasma; PT, physical therapy; SSI, subacromial steroid injection; SSNB, suprascapular nerve block; T0-T3, time zero (baseline)-time 3 (months).

**Table A5:** Associations Between Changes in Serum BDNF Levels and Clinical Outcomes (ΔT1-T0)*^*a*^*

Scale	PT	PT + SSI	PT + SSNB	PT + PRP
NRS	**<.01**	.95	.93	.06
LPS	**.04**	.32	.42	.49
PSQI	.52	.91	.33	.93
AIS	**<.01**	.52	.31	.46
MoCA	>.99	.14	.90	.97
TMT-A	.48	.32	.61	.45
TMT-B	*.05*	.70	.38	.10
ASES	.18	.26	.52	.57
SST	.97	.49	.59	.44
CMS	.70	.32	.42	.49

a Data represent *P* values from Spearman correlation between changes in BDNF and changes in clinical outcomes from T0 to T1. Bold values indicate significant results (*P* < .05); italicized values indicate marginally significant results (*P* = .05). AIS, Athens Insomnia Scale; ASES, American Shoulder and Elbow Surgeons; BDNF, brain-derived neurotrophic factor; LPS, Laitinen Pain Scale; MoCA, Montreal Cognitive Assessment; NRS, Numeric Rating Scale; PRP, platelet-rich plasma; PSQI, Pittsburgh Sleep Quality Index; PT, physical therapy; SSI, subacromial steroid injection; SSNB, suprascapular nerve block; T0-T1, time zero (baseline)-time 1 (month); TMT-A, Trail Making Test, Part A; TMT-B, Trail Making Test, Part B.
